# The Emerging Roles of Chromogranins and Derived Polypeptides in Atherosclerosis, Diabetes, and Coronary Heart Disease

**DOI:** 10.3390/ijms22116118

**Published:** 2021-06-06

**Authors:** Takuya Watanabe

**Affiliations:** Department of Internal Medicine, Ushioda General Hospital/Clinic, Yokohama, Kanagawa 230-0001, Japan; watanabe_9721@yahoo.co.jp; Tel.: +81-45-521-5147; Fax: +81-45-503-1609

**Keywords:** chromogranins, catestatin, vasostatin, pancreastatin, secretoneurin, atherosclerosis, diabetes, hypertension, coronary heart disease

## Abstract

Chromogranin A (CgA), B (CgB), and C (CgC), the family members of the granin glycoproteins, are associated with diabetes. These proteins are abundantly expressed in neurons, endocrine, and neuroendocrine cells. They are also present in other areas of the body. Patients with diabetic retinopathy have higher levels of CgA, CgB, and CgC in the vitreous humor. In addition, type 1 diabetic patients have high CgA and low CgB levels in the circulating blood. Plasma CgA levels are increased in patients with hypertension, coronary heart disease, and heart failure. CgA is the precursor to several functional peptides, including catestatin, vasostatin-1, vasostatin-2, pancreastatin, chromofungin, and many others. Catestatin, vasostain-1, and vasostatin-2 suppress the expression of vascular cell adhesion molecule-1 and intercellular adhesion molecule-1 in human vascular endothelial cells. Catestatin and vasostatin-1 suppress oxidized low-density lipoprotein-induced foam cell formation in human macrophages. Catestatin and vasostatin-2, but not vasostatin-1, suppress the proliferation and these three peptides suppress the migration in human vascular smooth muscles. Chronic infusion of catestatin, vasostatin-1, or vasostatin-2 suppresses the development of atherosclerosis of the aorta in apolipoprotein E-deficient mice. Catestatin, vasostatin-1, vasostatin-2, and chromofungin protect ischemia/reperfusion-induced myocardial dysfunction in rats. Since pancreastatin inhibits insulin secretion from pancreatic β-cells, and regulates glucose metabolism in liver and adipose tissues, pancreastatin inhibitor peptide-8 (PSTi8) improves insulin resistance and glucose homeostasis. Catestatin stimulates therapeutic angiogenesis in the mouse hind limb ischemia model. Gene therapy with secretoneurin, a CgC-derived peptide, stimulates postischemic neovascularization in apolipoprotein E-deficient mice and streptozotocin-induced diabetic mice, and improves diabetic neuropathy in db/db mice. Therefore, CgA is a biomarker for atherosclerosis, diabetes, hypertension, and coronary heart disease. CgA- and CgC--derived polypeptides provide the therapeutic target for atherosclerosis and ischemia-induced tissue damages. PSTi8 is useful in the treatment of diabetes.

## 1. Introduction

Coronary heart disease is now the leading cause of death worldwide [1]. The risk factors for coronary heart disease involve hypercholesteremia, diabetes, hypertension, obesity, and metabolic syndrome [1]. Coronary heart disease exhibits myocardial ischemia and dysfunction induced by significant stenosis in coronary arteries that supply the heart with blood [1]. It is usually caused by atherosclerosis, which is a chronic inflammatory disease with a buildup of cholesterol-rich plaques inside the artery walls [1]. Atherosclerosis is characterized by a complex multicellular process [2], and is triggered by arterial injury-induced endothelial inflammation. This results in the formation of intimal atheroma and plaque caused by oxidized low-density lipoprotein (LDL)-induced macrophage foam cell formation, vascular smooth muscle cell (VSMC) proliferation, and extracellular matrix (ECM) production [2].

Chromogranin A (CgA), chromogranin B (CgB), and chromogranin C (CgC), which are abundantly expressed in neurons, endocrine, and neuroendocrine cells, are associated with carbohydrate metabolism [3]. These proteins belong to a class of granins, which were first defined as proteins involved in the formation and function of secretory granules [4,5,6]. The other part of the name (chromo-) relates to the fact that chromaffin granules were the first discovered to contain granin proteins [7]. CgA is known to play a significant role in the pathogenesis and development of type 1 diabetes, and is associated with its complications [8,9,10]. A CgA-derived peptide, pancreatin, is expressed in the pancreatic islet [11], and has a strong inhibitory action on insulin secretion from the islet β-cells [12]. A pancreastatin inhibitor can cancel the diabetogenic effects of pancreastatin [13].

Previous studies have shown that genetic polymorphisms of CgA, CgB, and CgC are associated with hypertension [14,15,16]. A recent study suggests that a common genetic variant of the CgA-derived peptide catestatin is associated with hypertension and atherogenesis [17,18]. In addition to catestatin, the other CgA-derived peptides, vasostatin-1 and vasostatin-2, exert atheroprotective effects [19,20,21]. Both catestatin and vasostatin-1 have vasorelaxant properties [22]. Vasoconstriction-inhibiting factor (VIF) suppresses angiotensin II-induced vasoconstriction [23]. Thereby, catestatin, vasostatin-1, and VIF have counter-regulatory effects against hypertension [22,23].

This review introduces the emerging roles of CgA, CgB, CgC, and derived polypeptides in the multicellular pathogenesis and development of atherosclerosis, diabetes, and coronary heart disease. Using these proteins and polypeptides along with current challenges and advances in clinical practice, such as biomarker and therapeutic strategies for atherosclerotic cardiovascular diseases, will be discussed in this review.

## 2. Characteristics of CgA, CgB, and CgC

Granins form a family of highly acidic proteins that are primarily found in the lumen of dense-core secretory granules of endocrine cells and neurons [24]. The most abundant members of this family are CgA, CgB (also called secretogranin I), and CgC (also called secretogranin II) [3]. CgA was first isolated from chromaffin cells of the adrenal medulla [25], and CgB was initially characterized in a rat pheochromocytoma cell line [26]. CgC (secretogranin II) was independently discovered later, in the anterior pituitary and prostate cancer cells [27]. Human *CHGA*, *CHGB*, and *SCG2* loci have been mapped to chromosomes 14q32.12, 20pter–p12, and 2q35–2q36, respectively [28]. Human CgA and human CgB consist of 457 and 677 amino acids with molecular weights of 51 and 78 kDa, respectively [3,29,30]. CgA and CgB contain a homologous, disulfide-bonded loop structure near their termini and another homologous sequence at C-termini [29,31]. Human CgC is composed of 617 amino acids with a molecular weight of 68 kDa [3], and does not have the disulfide-bonded loop and the homologous C-terminal domain of CgA and CgB [32]. However, CgC contains a weak homology to the C-terminal region of CgA and CgB at a position of ~120 amino acid residues upstream from its C terminus [3]. When compared to genomic structures of CgC and CgB, it appears that CgC as a whole (except for its 27 amino acid signal peptide) corresponds to exon 4 of CgB [3].

CgA, CgB, and CgC are characterized by the following: (1) an abundance of acidic amino acids, (2) calcium binding sites, (3) multiple potential dibasic cleavage sites, (4) a multitude of post-translational modifications, and (5) the tendency to self-aggregate at low pH/high calcium conditions typical of secretory granules [3].

Both CgA and CgB regulate both catecholamine levels and blood pressure [33]. They also have an effect on glucose and insulin metabolism [10,34]. CgC induces dopamine release from the striatum [35].

## 3. Cgs-Derived Polypeptides

CgA was identified as an acidic protein costored and coreleased with ATP and catecholamines in chromaffin granules of neuroendocrine cells in the adrenal medulla [25]. CgA is also present in other secretory vesicles of neuronal and endocrine tissues including the pancreatic islet, in addition to keratinocytes, cardiomyocytes, ECs, and macrophages [3,19,20].

**Figure 1:**
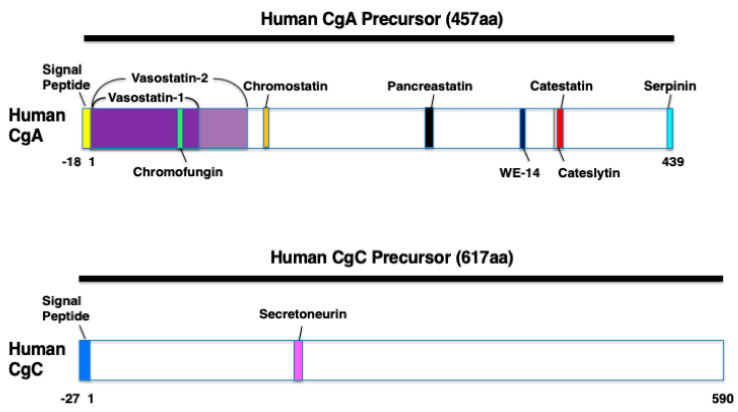
The domains of the various biologically active polypeptides derived from human chromogranin A (CgA) and human chromogranin C (CgC). Schematic diagrams showing vasostatin-1 (CgA1–76), vasostatin-2 (CgA1–113), chromofungin (CgA47–66), chromostatin (CgA124–143), pancreastatin (CgA250–301), WE-14 (CgA324–337), cateslytin (CgA344–358), catestatin (CgA352–372), serpinin (CgA402–439), and secretoneurin (SgII154–186).

Human *CHGA* spans 12,192 base pairs and originates in eight exons and seven introns [36]. The derived transcript of 2041 base pairs is translated into the 457 residues pre-CgA protein (51 kDa), including an 18 amino acid signal sequence [29,37]. The human mature CgA protein consists of 439 amino acids (49 kDa) and is characterized by 8–10 pairs of dibasic cleavage sites [36,37]. CgA can be proteolytically processed in various tissues and thereby serves as a precursor for several biological active peptides [36]. The cleavage of CgA at its dibasic sites is performed by intragranular and extracellular proteases, such as the following prohormone convertase 1 (PC1), PC2, furin, cysteine protease cathepsin L, the serine proteases plasmin and thrombin, and also by kallikrein [3,37]. On the basis of the cleavage sites, post-translational modifications (glycosylation and phosphorylation), and proteolytic processing, human CgA (457 amino acids) can result in nine biological active peptides including the following: vasostatin-1 (CgA1–76), vasostatin-2 (CgA1–113), chromofungin (CgA47–66), chromostatin (CgA124–143), pancreastatin (CgA250–301), WE-14 (CgA324–337), cateslytin (CgA344–358), catestatin (CgA352–372), and serpinin (CgA402–439) [36] (Figure 1). In addition, bovine CgA (449 amino acids) contains the two biological active peptides, VIF (CgA79–113) and chromacin (CgA173–194) [29]. However, VIF and chromacin have been recently demonstrated to be present in human blood and tissues [23,38].

CgB is abundantly expressed in many neurons and endocrine cells [3]. After synthesis, CgB is posttranslationally O-glycosylated and stored to large secretory vesicles [3]. Within granules, CgB is proteolytically processed at diabasic Lys-Arg and monobasic Arg sites to several proteins of intermediate size and small peptides [3]. From bovine CgB (646 amino acids), the 13-amino acid peptide secretolytin (CgB614–626) was identified, and has the biological activity as an antibacterial agent [39]. Secretolytin has been also found in human blood [40].

CgC is produced in the brain at the highest degree of >90% [41]. CgC is also abundantly expressed in spinal cord, skeletal muscle, and myocardium [41,42]. CgC is cleaved by the proteases PC1/3 and PC2 to the 33-amino acid peptide secretoneurin (SgII154–186) [42] (Figure 1). The activity of these proteases is increased three-fold in the failing myocardium [42]. Under pathophysiological conditions, such as hypoxia, secretoneurin expression is increased in the brain, skeletal muscle, and myocardium [41,42,43].

The roles of Cgs and their cleavage products as the biomarkers and pathogenesis of diabetes and atherosclerotic cardiovascular diseases are described in the following chapters.

## 4. Biomarker for Diabetes, Metabolic Syndrome, and Cardiovascular Disease

The half-life of CgA is relatively long; ~18.4 minutes in vivo in humans [44]. Circulating CgA concentrations are 25–100 ng/mL under normal conditions and increase under physio-pathological conditions [45]. In particular, plasma CgA concentrations increase by 12.5-fold in patients with pheochromocytoma compared with normal men [44].

CgA is known as an important biomarker of diabetes and cardiovascular diseases in addition to neuroendocrine tumors [38,46,47]. Higher levels of CgA in the circulating blood have been reported in patients with type 1 diabetes, but not those with type 2 diabetes, when compared with control subjects [47] (Table 1). Plasma levels of pancreastatin are higher in patients with type 2 and gestational diabetes compared with control subjects [48,49] (Table 1). In contrast, serum levels of vasostatin-2 are lower in type 2 diabetic patients than in nondiabetic controls [50]. Serum CgB levels are lower in patients with type 1 diabetes, but not those with type 2 diabetes, when compared with control subjects [47] (Table 1). There are no significant correlations between serum CgA and CgB levels [47]. Serum catestatin levels are decreased in patients with metabolic syndrome compared with patients in the control category [51].

Since CgA is even more stable compared with cathecholamines in the circulating blood, its plasma levels reflect the sympathetic tone and adrenomedullary system activity, which are altered in coronary artery disease (CAD), heart failure, and hypertension [37]. Circulating levels of CgA are increased and associated with the mortality of patients with CAD [52,53,54,55] (Table 2). In CAD patients, plasma CgA levels rise even higher in the presence of heart failure [55]. Plasma levels of vasostatin-1 are positively associated with carotid atherosclerosis [56]. In contrast, circulating levels of vasostatin-2 and catestatin are significantly decreased in patients with CAD compared with healthy control groups of patients [19,50,57,58] (Table 2). Serum levels of vasostatin-2 are also decreased in patients with ischemic chronic heart failure [59]. However, catestatin levels are increased at the onset of acute myocardial infarction, which is correlated with norepinephrine levels [60], and leads to adverse events [61]. In addition, the increase in catestatin levels also contributes to coronary collateral development and left ventricular remodeling [62,63,64]. Plasma levels of vasostatin-1 and secretolytin are increased in patients with coronary artery bypass graft surgery [40].

Plasma levels of CgA, catestatin, and pancreastatin are significantly increased in patients with hypertension compared with healthy control subjects [65,66,67] (Table 2). There are higher plasma levels of CgA, CgB, catestatin, VIF, and secretoneurin in patients with heart failure compared with healthy control groups of patients [23,42,68,69,70]. Serum levels of CgA and CgB are significantly higher in the presence of carcinoid heart disease among patients with neuroendocrine tumors [71]. Levels of plasma CgA are much higher in patients with dilated cardiomyopathy or hypertrophic cardiomyopathy than the levels in the healthy controls [72]. Plasma vasostatin-1 levels are increased in patients with Takayasu arteritis [73]. High levels of secretoneurin are associated with the increased risk of mortality in patients with heart failure, aortic stenosis, or those patients undergoing various cardiac surgeries [74,75,76].

CgA is detected at higher levels in the saliva of type 2 diabetic patients compared with healthy and nondiabetic subjects [77,78]. In patients with type 2 diabetes, the high levels of salivary CgA are associated with periodontal damage [78]. Therefore, CgA in saliva may be a biomarker for oral health in patients with type 2 diabetes. The levels of CgA, CgB, and CgC in the vitreous humor are higher in patients with diabetic retinopathy compared with nondiabetic subjects [79].

As clinical biomarkers, CgA, CgB, CgC, and derived polypeptides are closely associated with atherosclerotic cardiovascular diseases and diabetes. Next, this review describes their cardiovascular effects as well as the molecular and cellular mechanisms of their anti-atherosclerotic and anti-diabetic effects, and expands to their emerging roles in therapeutic strategies against atherosclerotic cardiovascular diseases and diabetes.

## 5. Cardiovascular Effects

Catestatin reduces blood pressure by inhibiting catecholamine secretion and stimulating histamine release [80]. Catestatin infusion directly dilates human blood vessels [81]. Catestatin and vasostatin-1 exert vasodilatory effects via nitric oxide (NO) release from vascular endothelial cells (ECs) [22]. Vasostatins and chromostatin suppress endothelin-1-induced vasoconstriction in human blood vessels [82,83]. VIF suppresses the vasoconstrictive effect of angiotensin II via AT2 receptor [23]. Pancreastatin activates Galpha16 and phospholipase C-β2 in myocardial membrane, suggesting that pancreastatin may regulate cardiac function [84]. Although chromofungin and chromacin have antimicrobial effects [85,86], their cardiovascular effects have not yet been elucidated. Serpinin enhances cardiac contractility (inotropy) via β-adrenergic receptors [87]. Cateslytin protects cardiomyocytes against lipopolysaccharide (LPS)-induced injury by decreasing inflammation and oxidized stress via toll-like receptor-4 [88]. Catestatin and the CgC-derived peptide secretoneurin stimulate ischemia-induced angiogenesis [89,90], but vasostatin-1 inhibits tumor angiogenesis and ocular neovascularization [91,92]. Secretoneurin protects against ischemic injury and apoptosis in the brain and skeletal muscle [42], and also improves cardiac dysfunction and inhibits cardiac remodeling following myocardial infarction [93].

In addition to cardiovascular protective effects, the atheroprotective effects of Cgs-derived polypeptides in vitro and in vivo are especially described in the next Chapter.

## 6. Atherosclerosis

**Figure 2:**
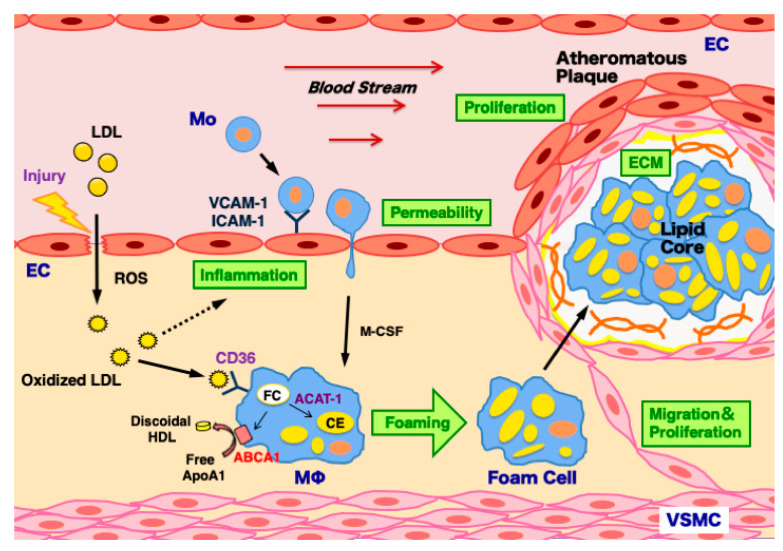
Mechanisms of atherosclerosis development in the arterial wall. Atherosclerosis is triggered by arterial injury-induced inflammation and hyperpermeability in endothelial cells (ECs). This process induces the infiltration of low-density lipoprotein (LDL) particles into the subendothelial space, and LDL is modified to oxidized LDL by reactive oxygen species (ROS), which further accelerates vascular inflammation. It stimulates monocyte adhesion to ECs via vascular cell adhesion molecule-1 (VCAM-1) and intercellular adhesion molecule-1 (ICAM-1) upregulation. Monocytes (Mo) infiltrate into the subendothelial space and then differentiate to macrophages (Mϕ) by macrophage colony stimulating factor (M-CSF). Macrophages uptake oxidized LDL via its receptor CD36 upregulation, and transform to foam cells via the decreased efflux of free cholesterol (FC) by the ATP-binding cassette transporter A1 (ABCA1) downregulation and increased cholesterol ester (CE) biosynthesis by acyl coenzyme A: cholesterol acyltransferase-1 (ACAT-1) upregulation. The massive accumulation of foam cells results in the formation of lipid core. To surround it, vascular smooth muscle cells (VSMCs) migrate, proliferate, and produce extracellular matrix (ECM), leading to the development of atheromatous plaques.

Atherosclerosis is triggered by arterial injury-induced inflammation. This process includes hyperpermeability, proliferation of ECs followed by the formation of atheromatous plaques involving oxidized LDL-induced foam cell formation in monocyte-derived macrophages, migration and proliferation of VSMCs, and extracellular matrix production by VSMCs [2] (Figure 2). As described above, in the formation and development of atherosclerosis in the arterial walls, three types of vascular cells, such as ECs, macrophage, and VSMCs, are known as the major players. Therefore, the effects of CgA- and CgC-derived polypeptides on these vascular cells are described in detail in the following sections.

### 6.1. ECs

Early atherosclerosis features vascular injury-induced changes in endothelial structure and barrier function that affect the traffic of molecules and solutes between the vessel lumen and the vascular wall [2]. Proatherogenic stimuli and cardiovascular risk factors, such as hypertension, dyslipidemia, diabetes, and smoking, increase endothelial permeability [94]. These factors share a common signaling denominator: an imbalance in the production/disposal of reactive oxygen species (ROS), broadly termed oxidative stress [94]. As a consequence of the activation of enzymatic systems leading to ROS overproduction, proatherogenic factors lead to a proinflammatory status that translates to changes in gene expression and functional rearrangements, including changes in the transendothelial transport of LDL [94]. Oxidation of LDL by ROS triggers the expression of adhesion molecules, such as vascular cell adhesion molecule-1 (VCAM-1) and intercellular adhesion molecule-1 (ICAM-1) in ECs [2]. Circulating monocytes attach to ECs and subsequently infiltrate into the intima [2]. In addition, EC proliferation contributes to the formation of intimal lesions [95]. The migration and proliferation of ECs are important phenomena for angiogenesis and also atherogenesis.

CgA, catestatin, vasostatin-1, and chromofungin suppress the permeability in ECs [91,96,97,98] (Table 3). Vasostatin-1 inhibits tumor necrosis factor-α (TNF-α)-induced gap formation in ECs [99], and also suppresses vascular endothelial growth factor (VEGF)-induced migration and proliferation of ECs [100] (Table 3). In contrast, secretoneurin activates transendothelial extravasation [101]. Catestatin and secretoneurin stimulate the migration and proliferation of ECs [89,102] (Table 3), contributing to angiogenesis. However, secretoneurin suppresses VEGF-induced EC proliferation [103] (Table 3).

Catestatin, vasostain-1, and vasostatin-2 suppress LPS- or TNF-α-induced expression of VCAM-1 and ICAM-1 in human ECs [19,20,50] (Table 3). Vasostatin-1 and vasostatin-2, but not catestatin, suppress LPS- or TNF-α-induced E-selectin expression, respectively [19,20,50]. Catestatin suppresses LPS-induced TNF-α expression [19], and vasostatin-1 suppresses LPS-induced expression of monocyte chemoattractant protein-1 (MCP-1) in human ECs [20]. Catestatin suppresses the adhesion of leukocytes to ECs [57]. Both vasostatin-1 and vasostatin-2 also suppress the adhesion of human monocytes to human ECs [20,50]. In contrast, secretoneurin stimulates the adhesion of human monocytes to human ECs [104]. Secretoneurin also upregulates basic fibroblast growth factor, platelet-derived growth factor-B, and VEGF, and activates NO synthase in ECs [93,102]. Secretoneurin induces endothelium-dependent relaxations in porcine coronary arteries [105].

### 6.2. Macrophages

Monocytes migrate into the subendothelial space, and then differentiate to macrophages [2]. Macrophages phagocytose oxidized LDL and transform into foam cells [2]. Foam cell formation depends on the homeostatic balance between the uptake of oxidized LDL via CD36, the efflux of free cholesterol controlled by the ATP-binding cassette transporter A1 (ABCA1), and cholesterol esterification by acyl coenzyme A: cholesterol acyltransferase-1 (ACAT-1) [106].

Catestatin and secretoneurin stimulate the migration of human monocytes [107,108]. These findings suggest that the two peptides contribute to the biodefence and inflammatory response in vascular walls. Catestatin and vasostatin-1 induce the anti-inflammatory phenotype and suppress the inflammation in human macrophages [19,20,109].

Catestatin and vasostatin-1 suppress oxidized LDL-induced foam cell formation in human macrophages [19,20] (Table 3). Macrophage foam cell formation by vasostatin-2 and pancreastatin has not yet been evaluated. Catestatin decreases ACAT-1 expression but increases ABCA1 expression without affecting CD36 expression in human macrophages [19]. Vasostatin-1 decreases both CD36 and ACAT-1 expression but increases ABCA1 expression in human macrophages [20]. Pancreastatin does not affect the expression of CD36, ACAT-1, and ABCA1 in human macrophages (Figure S1).

### 6.3. VSMCs

VSMCs contribute to the progression of atherosclerotic plaque through their migration, proliferation, and the production of ECM components, such as collagens, matrix metalloproteinases, fibronectin, and elastin. In particular, collagens promote the formation of the fibrous cap of atherosclerotic plaques [110]. The fibrous cap contributes to stabilizing atherosclerotic plaque to prevent its rupture. Elastin plays an essential role in the maintenance of vascular elasticity [111].

Catestatin, vasostatin-1, and vasostatin-2 suppress the migration of human VSMCs [19,20,112] (Table 3). Catestatin and vasostatin-2, but not vasostatin-1, suppress the proliferation of human VSMCs [19,20,112] (Table 3). In contrast, catestatin and secretoneurin promote the proliferation of VSMCs in rats [113,114] (Table 3).

In VSMCs, catestatin and vasostatin-1 suppress the expression of collagen-1 and collagen-3, respectively, and both peptides increase elastin expression [19,20] (Table 3). These findings suggest that catestatin and vasostatin-1 contribute to suppressing plaque progression and preserving vascular elasticity. Secretoneurin stimulates the expression of MCP-1 and VCAM-1 in VSMCs [115].

### 6.4. Murine Models of Atherosclerosis

The in vivo effects of CgA and its derived peptides on atherosclerosis have been evaluated in murine models with their exogenous infusion and endogenous deficiency. A chronic infusion of catestatin, vasostatin-1, or vasostatin-2 suppresses the development of atherosclerosis of the aorta in apolipoprotein E-deficient mice [19,20,21]. These anti-atherosclerotic effects are attributed to the molecular and cellular protective effects against atherosclerosis, as described above. Catestatin also attenuates insulin resistance, hypertension, and obesity in murine models, and contributes to the prevention of metabolic syndrome [116]. CgA-knockout mice reveal hypertension, high plasma catecholamine and adiponectin levels, and lower interleukin-6 and lipid levels compared with wild type mice [117]. CgA-knockout mice also exhibit enhanced insulin sensitivity despite obesity [118]. These findings suggest that CgA prevents the development of atherosclerosis. Next, the preventive effects of CgA and derived peptides on atherosclerotic cardiovascular diseases in murine models are described.

## 7. Myocardial Ischemia/Reperfusion Injury, Hind Limb Ischemia, and Stroke

CgA dilates coronary arteries and induces negative inotropic effects via Akt/NO/cGMP/protein kinase G pathway in hypertensive rat hearts [119]. Catestatin, vasostatin-1, and chromofungin protect ischemia/reperfusion-induced myocardial dysfunction via the NO-dependent pathway in rats [120,121,122]. Vasostatin-2 protects against ischemic heart failure in rats with myocardial infarction [59]. A CgA-derived peptide named pGlu-serpinin protects ischemia/reperfusion-induced myocardial dysfunction in normotensive and hypertensive rats [123]. Pretreated H9c2 cells (embryonic rat cardiomyocytes) with pGlu-serpinin are protected against hypoxia/reoxygenation [123]. Serpinin enhances myocardial contractility via β-adrenergic receptors followed by the adenylate cyclase/cAMP/protein kinase A pathway [87].

In addition, gene therapy with the CgC-derived peptide secretoneurin ameliorates hind limb and myocardial ischemia without influencing systemic atherosclerosis in apolipoprotein E-deficient mice [124]. Secretoneurin protects skeletal muscle and myocardium against ischemic injury and apoptosis [42]. Secretoneurin gene therapy has a variety of effects. It stimulates coronary angiogenesis, improves left ventricular function, and inhibits myocardial remodeling in a rat model of myocardial infraction [93]. Oral administration of secretoneurin enveloped in nanoparticles restores blood flow in the mouse hind limb ischemia model [125]. Secretoneurin gene therapy also stimulates postischemic neovascularization in streptozotocin-induced diabetic mice [126], and improves diabetic neuropathy in db/db mice [127]. Secretoneurin suppresses hypertrophy and oxidative stress via AMP-activated protein kinase (AMPK)/extracellular signal-regulated kinase (ERK) pathways in mouse cardiomyocytes [128]. Secretoneurin promotes neuroprotection and neuroplasticity via the Janus kinase-2/signal transducer and activator of transcription-3 pathway in murine models of stroke [129,130].

## 8. Diabetes

CgA is known to play a significant role in the pathogenesis and development of type 1 diabetes [10]. In vivo and in vitro experiments have determined that the function of CgB relates to the physiological secretion of insulin. CgB regulates early-stage insulin granule trafficking from the Golgi in pancreatic islet β-cells [34]. Catestatin suppresses hepatic glucose production and improves insulin sensitivity [131]. WE-14 (the abbreviation comes from N- and C-terminal amino acids and the length of the molecule) and human CgA10–19 serve as an autoantigen for both CD4+ and CD8+ β-cell-destructive diabetogenic T-cell clones in type 1 diabetes [132,133]. A recent study has identified a CgA29–42 peptide within vasostatin-1, an N-terminal natural derivative of CgA, as the BDC2.5 TCR epitope [134]. Having the necessary motif for binding to I-A(g7), it activates BDC2.5 T-cells and induces an interferon-γ response [134]. More importantly, adoptive transfer of naive BDC2.5 splenocytes activated with CgA29–42 peptide transferred diabetes into NOD/SCID mice [134].

Pancreastatin inhibits insulin secretion from pancreatic islet β-cells [12] and also regulates glucose, lipid, and protein metabolism in liver and adipose tissues [135]. Pancreastatin inhibits glucose uptake and glycogen synthesis but stimulates gluconeogenesis in hepatocytes [135]. Pancreastatin inhibits glucose uptake and glycogen synthesis in adipocytes [136]. Pancreastatin increases lipid droplets and ROS production in 3T3-L1 adipocyte cells [137]. These effects of pancreastatin are exerted via phosphatidylinositol 3-kinase/protein kinase C and glycogen synthase kinase-3 [136]. Pancreastatin plays a significant role in obesity-induced insulin resistance [138]. In healthy humans, a standard meal increases serum pancreastatin levels [139], and human pancreastatin infusion decreases forearm glucose uptake [140]. An intravenous infusion of human pancreastatin-16 suppresses the elevation of serum insulin levels without glucose overshoot on an oral glucose tolerance test in healthy humans [141]. Pancreastatin may induce the impaired insulin secretion and insulin resistance in the setting of diabetes and/or obesity.

Pancreastatin inhibitor peptide-8 (PSTi8), which consists of 21 amino acids (PEGKGEQEHSQQKEEEEEMAV-amide), exerts antidiabetic effects. These effects have been demonstrated by cell and animal studies [13,142,143,144]. PSTi8 decreases pancreastatin-induced insulin resistance in HepG2 cells (human liver cancer cells) and 3T3-L1 cells (mouse adipocyte cells) [13,142]. PSTi8 increases glucose uptake via enhanced glucose transporter type 4 in L6 cells (rat skeletal myoblast cells) [13,143] and decreases hepatic glucose release [144]. The treatment with PSTi8 increases insulin sensitivity in db/db, high fat and fructose-fed streptozotocin-induced insulin resistance mice [13]. PSTi8 improves the obesity-associated insulin resistance and inflammation in skeletal muscle [143], and improves hyperinsulinemia-induced obesity and inflammation-mediated insulin resistance in adipose tissue via inhibition of ERK/c-Jun N-terminal protein kinase pathways [137]. PSTi8 also improves dexamethasone-induced fatty liver by suppressing lipid deposition and oxidative stress through the glucose-regulated protein-78 followed by the AMPK pathway [144]. Further clinical studies are needed to clarify the efficacy of PSTi8 in the treatment of patients with diabetes and obesity.

## 9. Conclusions

CgA and derived polypeptides are the convincing biomarkers for atherosclerosis, diabetes, hypertension, and cardiovascular diseases. Circulating levels of CgA and pancreastain are high in type 1 and type 2 diabetes, respectively, because CgA is one of pathogeneses of type 1 diabetes, and pancreastatin induces insulin hyposecretion and insulin resistance. Circulating CgA levels are high in hypertension, CAD, and heart failure that show increments in the sympathetic tone and adrenomedullary system activity. Circulating levels of catestatin and vasostatin-2 are low in CAD. As catestatin and vasostatin-2 have atheroprotective effects, their decreased levels may be a risk factor for CAD.

PSTi8 is useful in the treatment of diabetes and metabolic syndrome. Catestatin, vasostatin-1, and vasostatin-2 serve the therapeutic target for atherosclerosis and coronary heart disease. Vasostatin-1 and secretoneurin stimulate ischemia-induced angiogenesis. Catestatin, vasostatin-1, and chromofungin protect ischemic myocardial damage. Cgs and derived polypeptides are a vision of new therapeutic strategies for atherosclerotic and ischemic cardiovascular diseases.

## Figures and Tables

**Table 1:** Circulating concentrations (ng/mL) of CgA, CgB, and pancreastatin in diabetes.

	Type 1 Diabetes	Control	*p* Value	Ref	Type 2 Diabetes	Control	*p* Value	Ref
CgA	61.64 ± 55.27 ↑	48.03 ± 19.99	0.0348	[47]	57.80 ± 34.74	49.97 ± 22.29	0.1587	[47]
CgB	89.39 ± 34.23 ↓	107.38 ± 59.77	0.0241	[47]	99.72 ± 54.79	112.54 ± 61.68	0.1698	[47]
Pancreastatin	NE	NE	NE	-	0.097 ± 0.022 ↑	0.026 ± 0.004	0.009	[48]

Data are shown as mean ± SD. NE = not examined.

**Table 2:** Circulating concentrations (ng/mL) of CgA, catestatin, vasostatin-2 in coronary heart disease (CHD) and hypertension.

	CAD	Control	*p* Value	Ref	Hypertension	Control	*p* Value	Ref
CgA	358 ± 304 ↑	108 ± 74	0.017	[52]	99.9 ± 6.7 ↑	62.8 ± 4.7	<0.001	[65]
Catestatin	2.09 ± 1.42 ↓	4.05 ± 3.52	0.0112	[19]	2.27 ± 0.83 ↑	1.92 ± 0.49	0.004	[66]
Vasostatin-2	4.45 ± 2.64 ↓	5.82 ± 3.22	<0.001	[50]	NE	NE	NE	-

Data are shown as mean ± SD. CAD = coronary artery disease, NE = not examined.

**Table 3:** Effects of CgA, catestatin, vasostatin-1, vasostatin-2, and secretoneurin on vascular cell responses for atherosclerosis.

	EC			Macrophage	VSMC			
	Permeability	Proliferation	VCAM-1	Foaming Cell	Migration	Proliferation	Collagen	Elastin
CgA	↓	NE	NE	NE	NE	NE	NE	NE
Catestatin	↓	↑	↓	↓	↓	↓	↓ (*1)	↑
Vasostatin-1	↓	↓	↓	↓	↓	→	↓ (*2)	↑
Vasostatin-2	NE	↑	↓	NE	↓	↓	NE	NE
Secretoneurin	↑	↑ or ↓	↑	NE	NE	↑	NE	NE

EC, endothelial cell; VSMC, vascular smooth muscle cell; NE, not examined. Arrows show these polypeptides-induced changes in each phenomenon. Catestatin and vasostatin-1 suppress the expression of collagen-1 (*1) and collagen-3 (*2), respectively.

## Data Availability

Not applicable.

## Supplementary Materials

The following are available online at https://www.mdpi.com/article/10.3390/ijms22116118/s1.

## Abbreviations

ABCA1	ATP-binding cassette transporter A1
ACAT-1	Acyl coenzyme A: cholesterol acyltransferase-1
AMPK	AMP-activated protein kinase
Cg	Chromogranin
CAD	Coronary artery disease
CE	Cholesterol ester
EC	Endothelial cell
ECM	Extracellular matrix
FC	Free cholesterol
ICAM-1	Intercellular adhesion molecule-1
LDL	Low-density lipoprotein
LPS	lipopolysaccharide
MCP-1	Monocyte chemoattractant protein-1
M-CSF	Macrophage colony stimulating factor
NO	Nitric oxide
PC	Prohormone convertase
PSTi8	Pancreastatin inhibitor peptide-8
ROS	Reactive oxygen species
TNF-α	Tumor necrosis factor-α
VCAM-1	Vascular cell adhesion molecule-1
VEGF	Vascular endothelial growth factor
VIF	Vasoconstriction-inhibiting factor
VSMC	Vascular smooth muscle cell
