# Supplementary figures and images for: The Emerging Roles of Chromogranins and Derived Polypeptides in Atherosclerosis, Diabetes, and Coronary Heart Disease
Source: Int J Mol Sci. 2021 Jun 6;22(11):6118. doi: 10.3390/ijms22116118 (PMC8201018; doi:10.3390/ijms22116118)

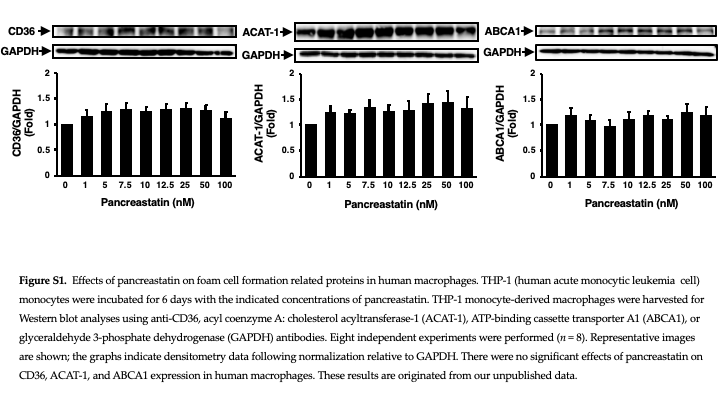

Supplement: Supplementary file 1 [file ijms-22-06118-s001.zip › ijms-1207515-supplementary.tiff]
